# Primary pure large cell neuroendocrine carcinoma of the urinary bladder: a case report and literature review

**DOI:** 10.3389/fonc.2024.1337997

**Published:** 2024-03-11

**Authors:** Zhenpeng Sun, Xin Liang, Changcun Zhang, Shizhang Song, Jiangang Gao

**Affiliations:** ^1^ Department of Urology, Qingdao Municipal Hospital, Qingdao, China; ^2^ Department of Urology, Yantai Yuhuangding Hospital Qingdao University, Yantai, China

**Keywords:** bladder cancer, large cell neuroendocrine carcinomas, radical cystectomy, toripalimab, prognosis

## Abstract

**Background:**

The large cell neuroendocrine carcinoma (LCNEC) of the urinary bladder is a rare malignancy. With its high aggressiveness and poor prognosis, the disease is often accompanied by metastasis or recurrence. The lack of specific clinical manifestations and imaging features causes considerable challenges for clinical diagnosis and treatment.

**Case presentation:**

We report a case of LCNEC of the urinary bladder. The patient was a 79-year-old male admitted to our hospital with recurrent episodes of asymptomatic gross hematuria. Based on the computed tomography (CT) scan findings, our patient presented with a bladder mass displaying invasion into the serosal layer, suggestive of muscle involvement and indicative of malignancy. The patient received a radical cystectomy, and the postoperative pathology confirmed primary, pure LCNEC of the urinary bladder. We gave him 16 cycles of toripalimab immunotherapy. As of follow-up, the patient was alive, and periodic CT reexamination showed no evidence of recurrence.

**Conclusions:**

We reviewed domestic and foreign literature and found no explicit treatment protocols exist for the disease. Surgical resection combined with chemotherapy were the most common treatments. Herein, we reported the first case of primary, pure LCNEC of the urinary bladder treated by radical cystectomy combined with pure immunotherapy, achieving sustained remission, which provides a new idea for the immunotherapy and integrative treatment of the disease.

## Introduction

Bladder cancer (BC) is a prevalent malignancy affecting the urinary tract and ranks as the ninth most common cancer globally, exhibiting significant morbidity and mortality rates (1). BCs encompass various histological subtypes, such as urothelial carcinomas (UCs), squamous cell carcinomas (SCCs), adenocarcinomas, and neuroendocrine tumors (2). However, primary bladder neuroendocrine carcinoma (BNEC) is a rare occurrence, constituting less than 1% of all urinary BCs (3). Among BNECs, the majority are classified as small cell neuroendocrine carcinomas (SCNEC), while large cell neuroendocrine carcinomas (LCNEC) are comparatively less prevalent (4, 5). LCNEC of the urinary bladder is characterized by a high degree of invasiveness. It is commonly identified at advanced stages during the initial diagnosis, leading to a significant probability of metastasis and an unfavorable prognosis (6). Moreover, due to its low incidence, absence of distinct clinical symptoms, and nonspecific imaging characteristics, it is often misidentified as urothelial carcinoma of the bladder. Currently, research and reports on it are extremely limited, and its diagnosis is primarily based on pathologic examination. Additionally, there is no standard protocol for the treatment of LCNEC of the urinary bladder. According to previous reports, the disease was commonly treated with a combination of surgery and chemotherapy (7).

We reported an elderly male patient with primary, pure LCNEC of the urinary bladder who underwent surgery and immunotherapy with temporary benefits. The aim was to emphasize the necessity of surgical treatment of LCNEC of the urinary bladder and to explore the possibilities for immunotherapy. In addition, this article conducted a comprehensive search of the Pubmed, Web of Science, and CNKI databases using the specific keywords “bladder” and “large cell neuroendocrine carcinoma”. We conducted a comprehensive review of the Chinese and English literature regarding LCNEC of the urinary bladder, especially case reports. And focused on examining the diagnostic approach and subsequent therapeutic strategies employed for this particular condition.

## Case presentation

**Figure 1:**
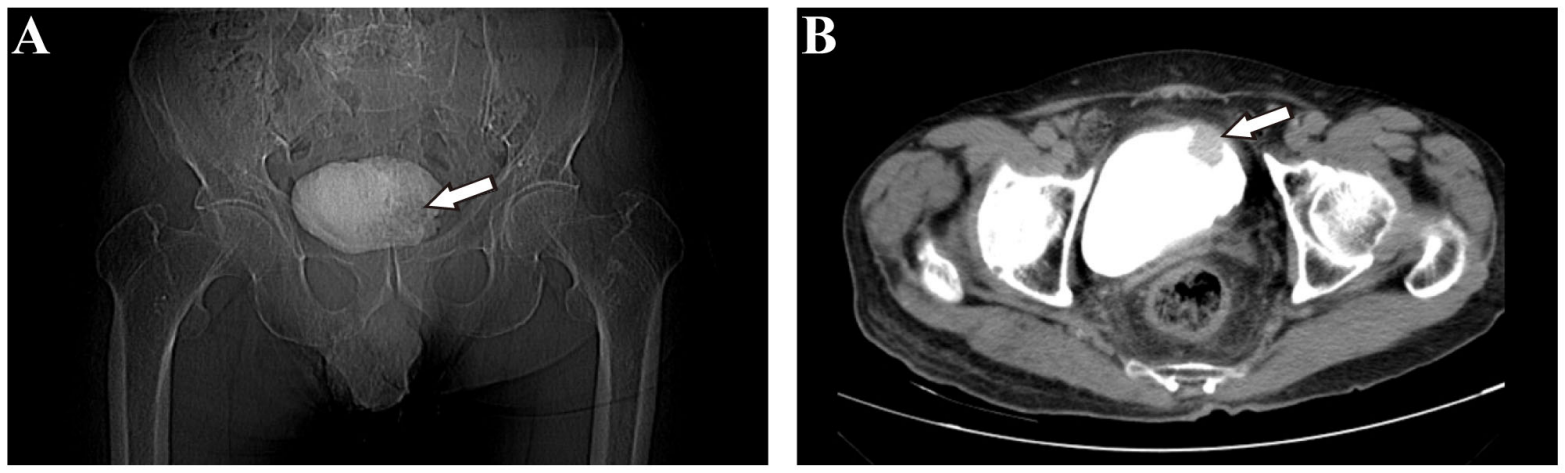
Imaging examination of patients before operation. **(A)** Coronal view of pelvic X-ray film revealed a shadow on the left bladder wall (white arrow). **(B)** Axial view of pelvic enhanced computed tomography (CT) revealed a cauliflower-like space-occupying lesion in the left anterior wall of the bladder.

**Figure 2:**
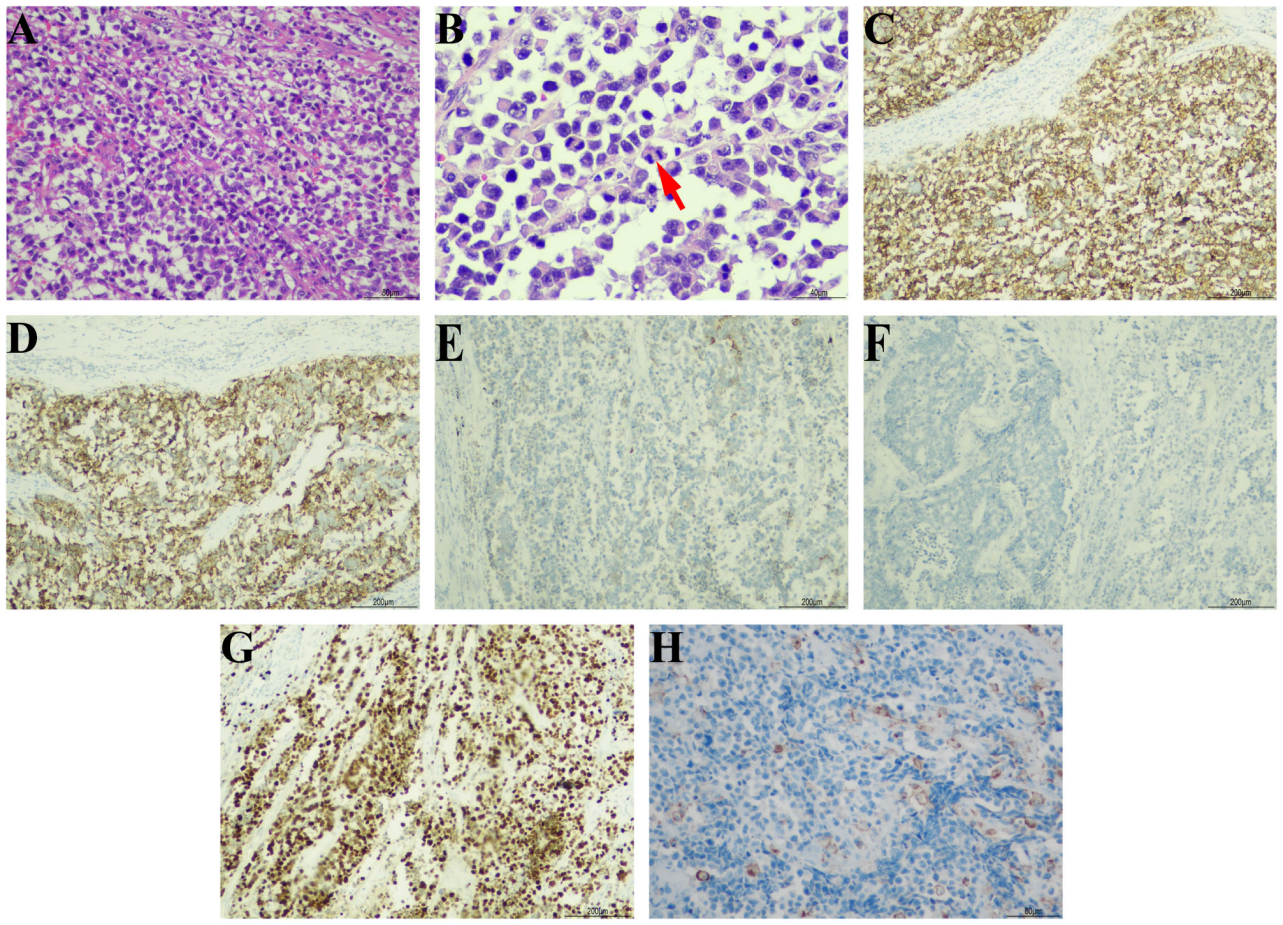
Pathological morphology and immunohistochemical results. **(A)** The tumor cells showed pleomorphism and invasive distributions (hematoxylin-eosin, ×200). **(B)** Typical mitotic phase can be seen in tumor cells (red arrow) (hematoxylin-eosin, ×400). The immunohistochemical staining of tumor cells showed **(C)** broad positivity for CD56 (×100), **(D)** diffuse positive expression of synaptophysin (×100), **(E)** focal positive expression of CK7 (×100), **(F)** negative expression of CgA (×100), **(G)** wildly positive expression of Ki-67 (×100), and **(H)** local positive rate of PD-L1 stain (×200).

On September 7th, 2021, a 79-year-old male was admitted to our hospital with recurrent episodes of asymptomatic gross hematuria. Prior to admission, the patient had experienced one month of asymptomatic gross hematuria that was brilliant red and accompanied by black blood clots. There was no percussive pain in either kidney, no tenderness in bilateral ureter areas, and no tenderness in the bladder area. The patient’s history of hypertension dated back two years. His enhanced computed tomography (CT) scan conducted last month revealed a mass that had invaded the serous membrane layer on the left bladder sidewall (**Figures 1A, B**). There was a high probability of lymph node involvement and an old renal infarction. Following hospitalization in that September, a chest CT was conducted to stage and evaluate the tumor, but no evidence of distant metastasis was detected. The preoperative urine routine tests demonstrate a significant abundance of red and white blood cells (**Supplementary Table 1**). The preoperative evaluations of liver and kidney function indicate a marginal increase in creatinine concentrations (**Supplementary Table 2**). A wide basal cauliflower-like tumor ~5 cm in diameter was observed on the left wall of the bladder by cystoscopy. Following the acquisition of informed permission from the patient, a radical cystectomy procedure with lymph node dissection was conducted in September 2021. Urinary diversion was performed via ureteral cutaneous ostomy due to the patient’s advanced age. The tumor primarily consisted of large pleomorphic cells with moderate cytosolic volume and coarse nuclear chromatin, arrayed in trabecular and rosette patterns, as disclosed by microscopic examination (**Figures 2A, B**). The average mitotic count was about 20 per 10 high-power fields. Additional pathology H&E images showcasing a magnified view of the large pleomorphic cells with mitosis can be accessed in **Supplementary Figure 1**.

The immunohistochemical examination was performed using a DAKO auto stainer (DAKO Coverstainer, Germany), and detailed information regarding the corresponding antibodies can be found in **Supplementary Table 3**. The result revealed broad positivity for cytokeratin (CK), CD56 (**Figure 2C**), and synaptophysin (**Figure 2D**), as well as localized positivity for CD138 and CK7 (**Figure 2E**). The results for GATA3, NSE, HER2, and CgA (**Figure 2F**) were found to be negative. The Ki-67 proliferation index was found to be greater than 90% (**Figure 2G**). The expression of PD-L1 by tumor cells was seen to be 3% (**Figure 2H**), whereas the expression by infiltrating immune cells was 1%. The tumor exhibited infiltration of the outer mold of the bladder, with several invasions of vascular, lymphatic arteries, and nerves. Additionally, it displayed infiltration of the perivesical tissues and metastases in two right pelvic lymph nodes. There were no components of urothelial carcinoma or other histological subtypes such as small cell neuroendocrine carcinoma. The individual was ultimately diagnosed with primary pure LCNEC of the urinary bladder (pT_3_N_2_M_0_).

**Figure 3:**
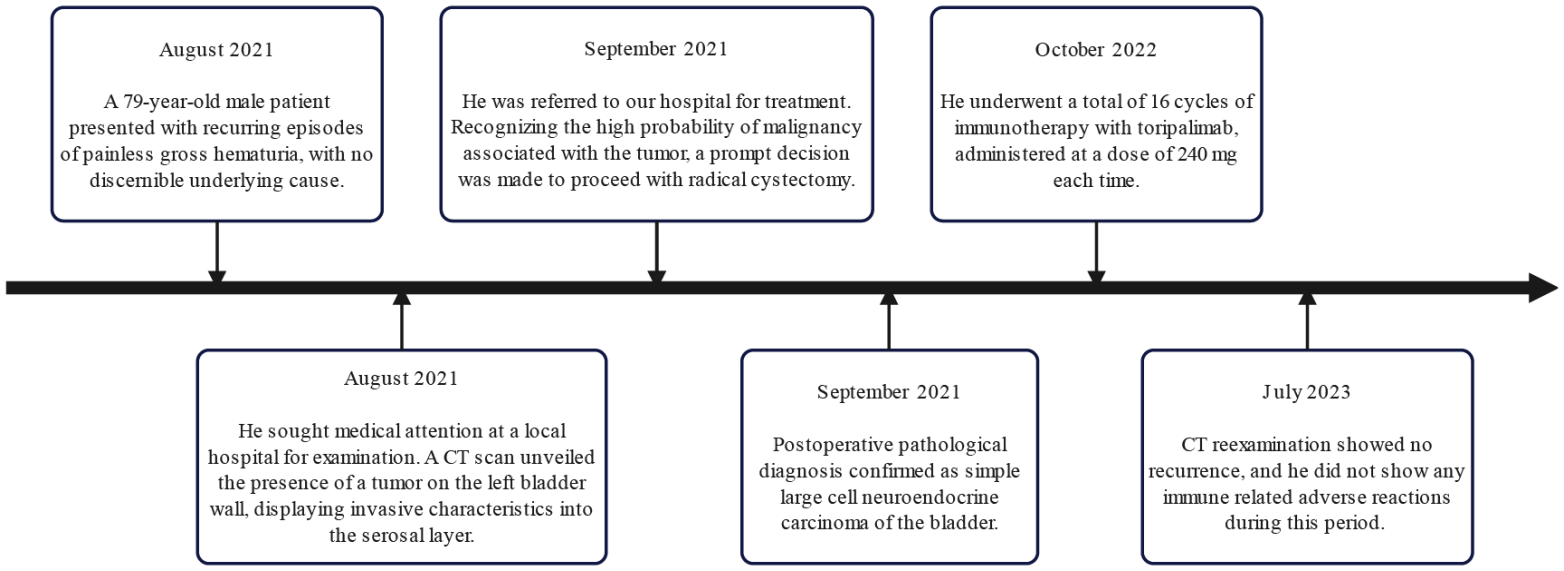
The timeline from disease discovery to patient diagnosis and treatment.

Considering the rarity of the disease and unclear treatment guidelines, a multidisciplinary team discussed the best treatment approach to prevent the recurrence of the disease. The consensus was treatment with chemotherapy using carboplatin and etoposide. Nevertheless, the patient and his family declined the chemotherapy treatment. As an alternative, 16 cycles of immunotherapy with toripalimab 240 mg were administered, and CT reexaminations were conducted every 3 months. His final CT reexamination in July 2023 revealed no recurrence, and he did not exhibit any immune-related adverse reactions during the two years after the primary diagnosis. **Figure 3** depicts the onset, examination, and treatment schedule for this patient.

## Discussion

The tissue origin of BNEC remains uncertain, and a few explanations have been proposed: ① pluripotent urothelial stem cells; ② pre-existing neuroendocrine/enterochromaffin cell population in the submucosa or in normal urothelium; ③ metaplasia of urothelium; and ④ transformation of urothelial carcinoma cells (5). The mainstream viewpoint favors stem cell origins, whose capacity to differentiate into various cell types explains the heterogeneity of bladder NEC and the expression of distinct neuroendocrine and epithelioid markers (8). BNEC, including LCNEC, typically co-occurs with urothelial carcinoma, squamous cell carcinoma, and adenocarcinoma, indicating a possible common clonal origin for these carcinomas (6, 9–12).

LCNEC was first identified in the lung and typically found in the respiratory and digestive tracts. Primary LCNEC of the urinary bladder is an exceedingly uncommon malignant tumor, with only 54 cases documented in the literature in detail (**Table 1**), of which 80% are males (4, 6, 7, 9, 11–47). Abenosa et al. (13) first reported LCNEC of the urinary bladder in 1986. Due to its rarity, there had been limited studies and reports on this specific condition. As the disease continued to being reported, individuals were gradually realizing that it possesses clinical features that are different from small cell neuroendocrine carcinoma. It was not until 2016 that the World Health Organization (WHO) recognized this pathological variant subtype of bladder cancer (48). The average age of onset is close to 60 years (7). The manifestation of gross hematuria accompanied by lower urinary tract irritation constitutes a characteristic symptom (49), characterized at the cellular level by insufficient differentiation and profound invasiveness. As a result of the absence of characteristic clinical symptoms during the initial phases of the illness, delayed diagnosis is common, resulting in a high occurrence of metastases and a poor prognosis (42). Out of the previously described instances of LCNEC of the urinary bladder, 65% of cases had a pathological staging of T3 or T4 (the tumor has infiltrated the serosal layer) (50), possibly owing to the absence of a discernible correlation between early lesions and the manifestation of typical hematuria symptoms. The present patient was an elderly male who was admitted with painless macroscopic hematuria and suspected of lymph node metastases at the time of the initial diagnosis.

**Table 1:** Cases of primary LCNEC of the urinary bladder published in literature.

Authors	Year	Number	Age	Sex	Treatment	Follow-up (Months)	Metastasis	Recurrence	Status at Follow up
Abenoza et all (13)	1986	1	55	M	RC, CT	30	Yes	Yes	Died with Metastasis and Recurrence
Hailemariam et all (14)	1998	1	73	M	RC, LND	2	Yes	Yes	Died with Metastasis and Recurrence
Evans et all (15)	2002	1	82	M	TURBT, PC, RT	24	No	No	Alive without Recurrence
Dundr et all (16)	2003	1	54	F	RC, CT	16	Yes	Yes	Alive with Metastasis and Recurrence
Li et all (17)	2004	1	61	M	RC	8	No	No	Alive without Recurrence
Quek et all (18)	2005	5	61-79	M/F=4/1	RC or (RC, CT)	NA	Four with Metastasis	NA	Four Died with Metastasis and One Alive without Metastasis or Recurrence
Lee et all (19)	2006	1	32	M	TURBT, PC, CT	12	Yes	Yes	Alive with Metastasis and Recurrence
Alijo Serrano et all (20)	2007	1	40	M	RC, CT	13	NO	NO	Alive without Recurrence
Alijo Serrano et all (20)	2007	1	43	F	RC, RT	12	Yes	NA	Died with Metastasis
Akamatsu et all (4)	2008	1	63	M	RC, CT	16	NO	NO	Alive without Recurrence
Bertaccini et all (21)	2008	1	37	NA	RC, LND, CT	22	NO	NO	Alive without Recurrence
Lee et all (22)	2009	1	19	M	PC, CT	14	Yes	Yes	Died with Metastasis and Recurrence
Oshiro et all (23)	2010	1	76	F	PC	48	NO	NO	Alive without Recurrence
Tsugu et all (24)	2011	1	74	M	CT	5	Yes	NA	Died with Metastasis
Engles et all (25)	2012	1	65	M	RC, LND, CT	3	No	NO	Alive without Recurrence
Coelho et all (6)	2013	1	79	M	PC	3	No	No	Died of worsening of the general status
Coelho et all (6)	2013	1	37	M	TURBT	0.5	Yes	NA	Died of liver failure
Hata et all (11)	2013	1	84	M	TURBT	8	No	No	Alive without Recurrence
Colarossi et all (12)	2013	1	53	F	RC, LND, CT	7	Yes	NA	Died
Sari et all (26)	2013	1	67	M	TURBT	0.5	No	NA	Died of heart faiure
Mačák et all (27)	2013	1	88	M	TURBT	NA	No	NA	NA
Mačák et all (27)	2013	1	66	M	TURBT, CT	NA	Yes	NA	NA
Jaggon et al (28)	2013	1	59	M	TURBT	NA	Yes	NA	NA
Vasconcellos et all (29)	2013	1	63	F	TURBT	NA	Yes	NA	NA
Pusiol et all (30)	2013	1	68	M	RC, LND, CT	30	Yes	Yes	Died with Recurrence and widely Metastasis
Treglia et all (31)	2014	1	84	M	TURBT, CT	NA	No	NA	NA
Pusiol et all (32)	2014	1	68	M	RC, LND	NA	NA	NA	NA
Jiang et all (33)	2015	1	62	M	RC	NA	No	NA	NA
Jiang et all (33)	2015	1	71	M	RC	1	Yes	NA	Died with Metastasis
Jiang et all (33)	2015	1	70	M	TURBT	7	Yes	NA	Died with Metastasis
Radović et all (7)	2015	1	58	M	RC	5	Yes	NA	Died of deterioration of general condition
Gupta et all (34)	2015	5	Mean=71.8	M/F=4/1	NA	NA	No	Three with Recurrence	Three Died of Recurrence and Two Died of others
Chong et all (35)	2017	1	72	M	RC, CT	36	No	Yes	Alive with spontaneous remission
Zakaria et all (36)	2017	1	72	M	RC, CT, IT	11	Yes	NA	Died with Metastasis
Akdeniz et all (37)	2018	1	45	M	TURBT, CT, RT	NA	No	NA	NA
Goret et all (38)	2020	1	70	M	TURBT	NA	Yes	NA	NA
Halabi et all (39)	2020	1	64	M	TURBT, CT, RT	NA	No	Yes	Died with Recurrence and Metastasis
Xia et all (40)	2020	1	39	M	RC, LND, CT	59	No	No	Alive without Recurrence or Metastasis
Li et all (41)	2020	1	30	M	PC, CT	24	NA	No	Alive without Recurrence
Pini et all (9)	2021	1	49	M	TURBT, RC, LND, CT	26	No	Yes	Alive with Recurrence and Metastasis
Tlili et all (42)	2021	1	49	M	TURBT, CT, RT	12	Yes	NA	Alive with Metastasis
Lopedote et all (43)	2022	1	82	F	TURBT, CT, RT	12	No	Yes	Alive with Recurrence and Metastasis
Mahmoudnejad et all (44)	2023	1	65	F	TURBT, CT, BCG, RT	16	Yes	No	Alive without Recurrence or Metastasis
Xiao et all (45)	2023	1	66	M	NA	10	NA	No	Alive without Recurrence
He et all (46)	2023	1	67	M	TURBT, RC, LND, CT	39	NA	No	Alive without Recurrence
Mohanty et all (47)	2023	1	56	F	RC, CT	6	NA	NA	NA
Total	NA	54	NA	NA	NA	NA	NA	NA	NA

BCG, intravesical Bacillus Calmette-Guerin; CT, chemotherapy; F, female; IT, immunotherapy; LND, lymph node dissection; NA, not available; M, male; PC, partial cystectomy; RC, radical cystectomy; RT, radiotherapy; TURBT, trans-urethral resection of the bladder tumor.

The primary symptom of LCNEC of the urinary bladder is asymptomatic hematuria, which is not a specific clinical manifestation, making an accurate diagnosis challenging. Urinary CT is used for early LCNEC of the urinary bladder screening, and pelvic CT typically reveals a hypointense shadow. Contrast-enhanced CT facilitates the visualization of a low-density-filling defect within the bladder, and magnetic resonance imaging can also be utilized. In conjunction with positron emission tomography computed tomography (PET-CT), these imaging techniques help allow for the detection of distant metastases (51–53). However, a definitive diagnosis requires cystoscopy or postoperative pathology. According to the CT scan results, our patient presented with a bladder mass that had invaded the serosal layer, suggesting muscle involvement and a high likelihood of being cancerous. The cystoscopy revealed a tumor with a cauliflower-like form, including a wide base. The morphology of this specific type corresponds to the distinctive traits displayed by malignant tumors.

It was first recorded in the lung by Travis that the identification of pathological features of LCNEC (54), including the presence of large cells with a low nucleo-cytoplasmatic ratio, dense nuclear chromatin, prominent nucleoli, a high mitotic ratio exceeding 10 mitoses per 10 high-power fields, and the presence of immunohistochemical or ultrastructural evidence indicating neuroendocrine differentiation. Furthermore, in lung LCNEC, the chromatin typically exhibits variably coarse, granular, or vesicular, and is frequently accompanied by necrosis. In the distinction from UC, the sensitivity of synaptophysin, chromogranin A, and CD56 markers to LCNEC was 96%, with a specificity of up to 100%. Additionally, a Ki-67 proliferation index greater than 40% exhibits a sensitivity of 80% and a specificity of 86% in LCNEC, while UC typically has a Ki-67 proliferation rate of up to 25% (55). The absence or punctate manifestation of CK7 in SCNEC aids in distinguishing it from LCNEC, where CK7 expression is either partial or total (56). Reviewing our case, it was seen that the tumor tissue predominantly consisted of big pleomorphic cells with a moderate cytosolic volume and coarse nuclear chromatin. The immunohistochemical investigation revealed localized expression of CK7 and widespread expression of CD56 and synaptophysin. The Ki-67 proliferation index was found to be greater than 90%, suggesting a substantial level of cellular proliferation.

The infrequency of LCNEC of the urinary bladder has contributed to the lack of a standardized therapeutic approach, while the usual course of action involves radical cystectomy along with chemotherapy. Five patients with pure LCNEC of the urinary bladder were treated with radical cystectomy by Soundak Gupta and his colleagues. One of the cases was accompanied by distant metastasis, and the patient’s survival duration was 2.4 months. The remaining four instances did not exhibit any lymph nodes or distant metastases. A single patient who had chemotherapy treatment survived for a duration of 116.4 months. The mean survival duration for three patients who only underwent surgical intervention was 14.4 months. In addition, radical cystectomy was conducted in six instances of mixed LCNEC without the presence of distant metastases. The survival duration was 86.8 months for three patients who underwent combination chemotherapy, while it was 38.8 months for three patients who did not undergo combined chemotherapy (34). A recent study revealed that individuals with pure cancer had a poorer rate of survival compared to those with mixed cancer. Additionally, it was shown that performing radical resection after neoadjuvant chemotherapy enabled long-term survival in patients with localized LCNEC (55). However, the efficacy of immunological drugs in treating this urinary bladder condition remains undocumented.

In the realm of bladder LCNEC, the application of immunotherapy introduces a promising dimension to the therapeutic landscape. Immunotherapy, a rapidly evolving treatment paradigm, has demonstrated remarkable success across various cancer types, reshaping treatment outcomes. By augmenting the immune system’s inherent capabilities, immunotherapy employs diverse strategies, including the introduction of cytokines and antibodies (passive immunotherapy) or the administration of vaccines and immune cells (active immunotherapy) (57).

The intricate interplay between tumor cells and the tumor-infiltrating lymphocytes plays a pivotal role in disease progression. CD8+ T cells are capable of generating substantial quantities of anti-tumor cytokines and cytotoxic molecules, renowned for their exceptional anti-viral and anti-tumor capabilities (58). Evidence has verified a strong correlation between elevated levels of CD8+T cells and enhanced prognosis of neuroendocrine tumors (59). However, within the immune microenvironment of tumor tissue, there exists an immune escape mechanism that serves to evade the recognition and elimination of lymphocytes (60). Among the key players in this evasion strategy is PD-1, also known as CD279 (61). The interaction between PD-1 and its ligands, PD-L1 and PD-L2, serves as a critical checkpoint, exerting inhibitory effects on the activation and function of T cells. When this immune checkpoint block is destroyed, it will unleash the immune response, enabling T cells to recognize and eliminate cancer cells (62, 63). In addition, CTLA-4, another immune checkpoint, serves a crucial function as a negative regulatory element in the control of T cell activation (64). Prior research has discovered that CTLA-4 on Treg cells reduces the expression of CD80/CD86 on DCs by means of endocytosis, thus suppressing DC-induced T cell activation (65). It means that patients diagnosed with neuroendocrine tumors are anticipated to benefit from the application of targeted immune checkpoint anti-tumor immunotherapy.

Immunotherapy research, particularly the implementation of immune checkpoint inhibitors (ICIs), has significantly transformed the treatment of malignant tumors. In a retrospective study on patients with LCNEC, Naganuma et al. (66) demonstrated the efficacy and safety of PD-L1 inhibitors. Moreover, Sherman et al. (67) reported that LCNEC patients with low PD-L1 expression may also benefit from immunotherapy. Notably, according to a case report (68), immunotherapy was efficacious even for a LCNEC patient with negative PD-L1 expression. In addition to the level of PD-L1 expression, researchers have proposed a potential association between the effectiveness of immunotherapy in LCNEC and the tumor mutational burden (TMB). These findings contribute to our understanding of immunotherapy as a viable therapeutic approach for LCNEC. In a previous study, Zakaria et al. were the first to document the utilization of pembrolizumab, a PD-L1 inhibitor, as an immunotherapeutic for treating LCNEC of the urinary bladder with metastasis. Regrettably, the patient’s prognosis following the original diagnosis was just eleven months, due to a significant deterioration in both physical health and general quality of life.

Toripalimab, a PD-1 inhibitor developed independently, is the first medicine of its kind to receive approval for commercialization in China (69). Despite its considerable potential for treating BCs, the use of this specific medication for this condition has not been widely implemented thus far, unlike Pembrolizumab and Nivolumab (70). Ming Lu et al. (71) conducted a 20-month multi-center experiment to assess the effectiveness of toripalimab as a treatment intervention for patients with neuroendocrine neoplasms (NEN). Forty patients diagnosed with either current or metastatic NEN were included in the research. Out of these patients, eight experienced partial responses, while six showed persistent diseases. The trial produced an objective response rate (ORR) of 20%, a disease control rate (DCR) of 35%, and a median duration of response (DOR) of 15.2 months. In addition, patients with PD-L1 expression of 10% or above or a high tumor TMB had a greater objective response rate (ORR) in comparison to those with low PD-L1 expression of less than 10. In our specific case, the patient underwent a surgical surgery and then got toripalimab immunotherapy as an alternative treatment, without receiving chemotherapy. During regular post-treatment evaluations, the patient exhibited no indications of tumor recurrence or significant adverse events associated with the immune system.

In the intricate landscape of LCNEC of the urinary bladder, the pertinence of immune checkpoints and their modulation through immunotherapy assume paramount significance. LCNEC, with its unique immunological profile, may be predisposed to immune evasion, thereby rendering the blockade of immune checkpoints an enticing therapeutic avenue. Nevertheless, the complex mechanisms involved in immunotherapy, specifically the effectiveness of immune checkpoint inhibitors in LCNEC, have not been comprehensively assessed, and further investigation is required to discover breakthroughs in survival.

## Conclusion

LCNEC of the urinary bladder is a rare malignancy characterized by nonspecific clinical manifestations and a dismal prognosis. At present, there is no established standard treatment regimen, and surgical resection represents the primary approach. In this article, we present the first case of primary, pure LCNEC of the urinary bladder with lymph node metastasis that was successfully treated with radical cystectomy in conjunction with toripalimab, a form of ICIS, achieving sustained remission. Given the paucity of cases at present, more cases are warranted to help ascertain the underlying mechanisms and progression of LCNEC of the urinary bladder, as well as to develop more efficacious comprehensive treatment and management strategies.

## Patient’s statement

In this article, we reported the first case of primary, pure LCNEC of the urinary bladder with lymph node metastasis that was successfully treated with radical cystectomy in conjunction with toripalimab, a form of ICIS, achieving sustained remission. LCNEC of the urinary bladder is an extremely uncommon condition, characterized by nonspecific clinical manifestations and a dismal prognosis. At present, there is no established standard treatment regimen, and surgical resection represents the primary approach. Administering immunosuppressants for treatment at an early stage may lead to improved prognosis for patients. Given the paucity of cases at present, more cases are warranted to help ascertain the underlying mechanisms and progression of LCNEC of the urinary bladder, as well as to develop more efficacious comprehensive treatment and management strategies.

## Data availability statement

The original contributions presented in the study are included in the article/**Supplementary Material**. Further inquiries can be directed to the corresponding author.

## Ethics statement

Written informed consent was obtained from the individual(s) for the publication of any potentially identifiable images or data included in this article.

## Author contributions

ZS: Writing – review & editing, Writing – original draft, Visualization, Data curation, Conceptualization. XL: Writing – original draft, Supervision, Conceptualization. CZ: Writing – original draft, Supervision, Conceptualization. SS: Writing – original draft, Visualization, Methodology, Investigation. JG: Writing – review & editing, Visualization, Investigation, Conceptualization.
