# Supplementary figures and images for: Primary pure large cell neuroendocrine carcinoma of the urinary bladder: a case report and literature review
Source: Front Oncol. 2024 Mar 11;14:1337997. doi: 10.3389/fonc.2024.1337997 (PMC10961446; doi:10.3389/fonc.2024.1337997)

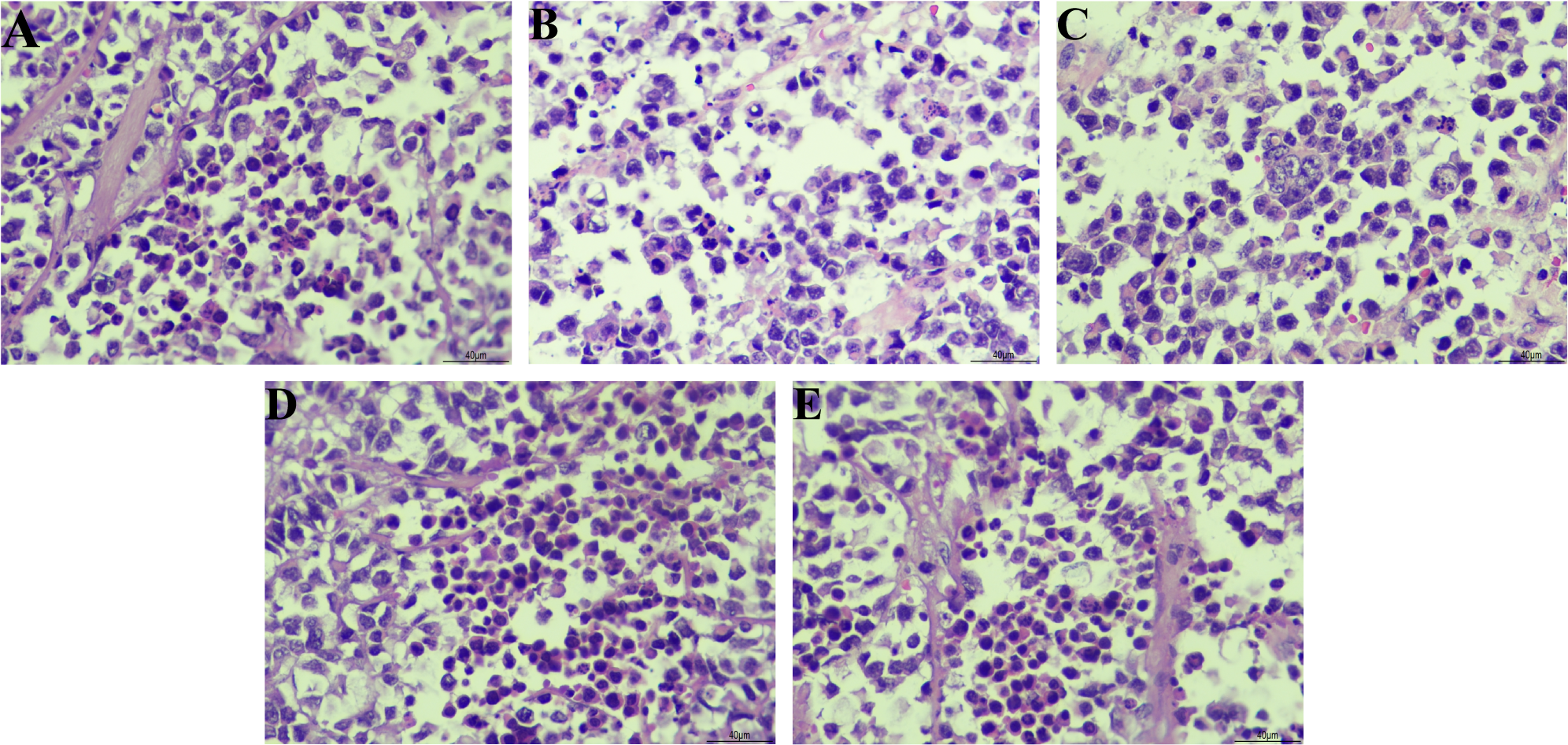

Supplement: Supplementary file 1 [file Image_1.tif]
