# Supplementary material for: Primary pure large cell neuroendocrine carcinoma of the urinary bladder: a case report and literature review
Source: Front Oncol. 2024 Mar 11;14:1337997. doi: 10.3389/fonc.2024.1337997 (PMC10961446; doi:10.3389/fonc.2024.1337997)
Supplement: Supplementary file 2 [file Table_1.docx]

**Supplementary Table 1. The results of urinalysis tests.**

|  | Value at presentation | Prompt | Reference | Unit |
| --- | --- | --- | --- | --- |
| COLOR | Faint yellow |  | Faint yellow |  |
| U-UBG | Normal |  | Weakly positive (+/-) |  |
| U-BIL | Negative |  | Negative |  |
| U-KET | Negative |  | Negative |  |
| U-BLD | +++ |  | Negative |  |
| U-PRO | + |  | Negative |  |
| U-NIT | Negative |  | Negative |  |
| WBC | 85.0 | ↑ | 0—25 | /ul |
| U-GLU | Negative |  | Negative |  |
| U-SG | 1.020 |  | 1.003-1.030 |  |
| PH | 5.5 |  | 4.5—8 |  |
| VitC | 0 |  | 0 | mmol/L |
| RBC | 644.7 | ↑ | 0—25 |  |
| TPC | 68 |  | 0--800 | /ul |
| PC | 0.00 |  | 0—0.34 | /ul |
| HYAL | 0.00 |  | 0—2 | /ul |
| SQEP | 0.65 |  | 0—28 | /ul |
| NSE | 0.65 |  | 0—6 | /ul |
| UNCX | 0.00 |  | 0—28 | /ul |
| YLC | 0.00 |  | 0—1 | /ul |
| MUCS | 3.27 |  | 0—28 | /ul |

U-UBG, urinary urobilinogen; U-BIL, urinary bilirubin; U-KET, urinary ketone body; U-BLD, urinary occult blood; U-PRO, urinary protein; U-NIT, urinary nitrite; WBC, white blood cell; U-GLU, urinary glucose; U-SG, urinary specific gravity; VitC, vitamin C; RBC, red blood cell; TPC, total plate count; PC, path cast; HYAL, hyaline cast; SQEP, squamous epithelial cell; NSE, Non squamous epithelial cells; UNCX, unclassified crystallization; YLC, yeast count; MUCS, mucous strands.
