# Supplementary material for: Primary pure large cell neuroendocrine carcinoma of the urinary bladder: a case report and literature review
Source: Front Oncol. 2024 Mar 11;14:1337997. doi: 10.3389/fonc.2024.1337997 (PMC10961446; doi:10.3389/fonc.2024.1337997)
Supplement: Supplementary file 3 [file Table_2.docx]

**Supplementary Table 2. The results of kidney and liver function tests.**

|  | Value at presentation | Prompt | Reference | Unit |
| --- | --- | --- | --- | --- |
| TP | 64.10 | ↓ | 65—85 | g/L |
| ALB | 34.50 | ↓ | 40—55 | g/L |
| GLO | 29.60 |  | 20--40 | g/L |
| A/G | 1.17 | ↓ | 1.2—2.4 |  |
| PAB | 243.00 |  | 200—430 | mg/L |
| TBIL | 11.90 |  | 0—26 | umol/L |
| DBIL | 3.00 |  | 0—4 | umol/L |
| IBIL | 8.90 |  | 0—17 | umol/L |
| TBA | 4.70 |  | 0—10 | umol/L |
| ALP | 102.14 |  | 45—125 | U/L |
| GGT | 15.78 |  | 10—60 | U/L |
| ALT | 11.11 |  | 9—60 | U/L |
| AST | 15.30 |  | 15—45 | U/L |
| CRE | 151.52 | ↑ | 57—111 | umol/L |
| UA | 232.01 |  | 208—428 | umol/L |
| CysC | 1.62 | ↑ | 0.59—1.03 | mg/L |
| ADA | 9.26 |  | 0—20 | U/L |
| AFU | 18.90 |  | 0—40 | U/L |
| BMG | 3.49 | ↑ | 1.3—3 | mg/L |
| GLU | 5.31 |  | 3.9—6.1 | mol/L |
| K | 3.73 |  | 3.5-5.3 | mol/L |
| NA | 142.00 |  | 137—147 | mol/L |
| CA | 2.24 |  | 2.11—2.52 | mol/L |
| P | 1.07 |  | 0.85—1.51 | mol/L |
| MG | 0.84 |  | 0.75—1.02 | mol/L |
| CO2 | 26.50 |  | 23—29 | mol/L |
| AG | 9.50 |  | 8—16 | mol/L |
| CL | 106.00 |  | 99-110 | mol/L |
| ALT/AST | 0.73 |  |  |  |
| SA | 651.00 |  | 400—700 |  |
| CG | 1.88 |  | 0—2.7 |  |
| BUN/CRE | 0.07 |  |  |  |
| BUN | 10.45 | ↑ | 3.6—9.5 | mmol/L |
| LAP | 44.00 |  | 30—70 | U/L |
| C1q | 132.20 | ↓ | 159—233 | mg/L |
| GLDH | 3.00 |  | 0—7 | U/L |

TP, total protein; ALB, albumin; GLO, globulin; A/G, albumin/ globulin; PAB, prealbumin; TBIL, total bilirubin; DBIL, direct bilirubin; IBIL, indirect red pigment; TBA, total bile acid; ALP, alkaline phosphatase; GGT, gamma glutamyltransferase; ALT, Alanine aminotransferase; AST, Aspartate aminotransferase; CRE, creatinine; UA, uric acid; CysC, cystatin-C; ADA, Adenosine deaminase; AFU, α-L-fucosidase; BMG, β2- microglobulin; GLU, glucose; K, kalium; NA, natrium; CA, calcium; P, phosphor; MG, magnesium; CO2, Magnesium carbon dioxide; AG, anion gap; CL, chlorine; SA, sialic acid; CG, cholyglycine; BUN, blood urine nitrogen; LAP, Leucine aminopeptidase; C1q, complement c1q; GLDH, glutamate dehydrogenase.
