# Supplementary material for: Primary pure large cell neuroendocrine carcinoma of the urinary bladder: a case report and literature review
Source: Front Oncol. 2024 Mar 11;14:1337997. doi: 10.3389/fonc.2024.1337997 (PMC10961446; doi:10.3389/fonc.2024.1337997)
Supplement: Supplementary file 4 [file Table_3.docx]

| Target | Antibody | Dilution | No. | Company | Contray |
| --- | --- | --- | --- | --- | --- |
| CD56 | NCAM1/CD56 Monoclonal antibody | 1:6000 | 60238-1-Ig | Proteintech | China |
| Synaptophysin | Synaptophysin Polyclonal antibody | 1:3000 | 17785-1-AP | Proteintech | China |
| CK7 | Cytokeratin 7-specific Polyclonal antibody | 1:4000 | 17513-1-AP | Proteintech | China |
| HER2 | HER2/ErbB2 Polyclonal antibody | 1:1000 | 18299-1-AP | Proteintech | China |
| PD-L1 | PD-L1/CD274 Monoclonal antibody | 1:15000 | 66248-1-Ig | Proteintech | China |
| CgA | Chromogranin A Monoclonal Antibody | 1:800 | MA5-13096 | Thermofisher | USA |
| GATA3 | GATA3 Antibody | 1:1500 | MA1-028 | Thermofisher | USA |
| NSE | NSE Polyclonal Antibody | 1:1000 | PA5-27452 | Thermofisher | USA |
| CD138 | Recombinant Anti-Syndecan-1 antibody [EPR6454] | 1:8000 | ab128936 | Abcam | UK |
| Ki-67 | Anti-Ki67 antibody | 1:2000 | ab15580 | Abcam | UK |

**Supplementary Table 3. Specific parameters of antibodies used in immunohistochemistry.**
